# Sensitivity of Electrocardiogram on Electrode-Pair Locations for Wearable Devices: Computational Analysis of Amplitude and Waveform Distortion

**DOI:** 10.3390/bios14030153

**Published:** 2024-03-21

**Authors:** Kiyoto Sanjo, Kazuki Hebiguchi, Cheng Tang, Essam A. Rashed, Sachiko Kodera, Hiroyoshi Togo, Akimasa Hirata

**Affiliations:** 1 Department of Electrical and Mechanical Engineering, Nagoya Institute of Technology, Nagoya 466-8555, Japan; k.sanjo.909@nitech.jp (K.S.); k.hebiguchi.290@nitech.jp (K.H.); kodera@nitech.ac.jp (S.K.); 2 Faculty of Information Science and Electrical Engineering, Kyushu University, Fukuoka 819-0395, Japan; tang@ait.kyushu-u.ac.jp; 3 Graduate School of Information Science, University of Hyogo, Kobe 650-0047, Japan; rashed@gsis.u-hyogo.ac.jp; 4 NTT Device Innovation Center, NTT Corporation, Atsugi 243-0198, Japan; hiroyoshi.togou.rs@ntt-tx.co.jp

**Keywords:** electrocardiogram, numerical human model, scalar-potential finite-difference method, wearable device

## Abstract

An electrocardiogram (ECG) is used to observe the electrical activity of the heart via electrodes on the body surface. Recently, an ECG with fewer electrodes, such as a bipolar ECG in which two electrodes are attached to the chest, has been employed as wearable devices. However, the effect of different geometrical factors and electrode-pair locations on the amplitude and waveform of ECG signals remains unclear. In this study, we computationally evaluated the effects of body morphology, heart size and orientation, and electrode misalignment on ECG signals for 48 scenarios using 35 bipolar electrode pairs (1680 waveforms) with a dynamic time warping (DTW) algorithm. It was observed that the physique of the human body model predominantly affected the amplitude and waveform of the ECG signals. A multivariate analysis indicated that the heart–electrode distance and the solid angle of the heart from the electrode characterized the amplitude and waveform of the ECG signals, respectively. Furthermore, the electrode locations for less individual variability and less waveform distortion were close to the location of electrodes V_2_ and V_3_ in the standard 12-lead. These findings will facilitate the placement of ECG electrodes and interpretation of the measured ECG signals for wearable devices.

## 1. Introduction

Cardiac arrhythmias and palpitations are commonly known symptoms associated with heart disorders. To assess and monitor such symptoms, the heart’s electrical activity can be visualized using an electrocardiogram (ECG), which is a widely used noninvasive diagnostic tool for cardiac diseases [1]. An ECG is obtainable via electrodes attached to the body surface. The well-known ECG electrode locations are the 12-lead configuration and body surface potential mapping (BSPM). The 12-lead ECG, which is primarily used to diagnose heart diseases in medical institutions, comprises six electrodes attached to the chest and three to the limbs. The direction of the leads is expanded to 12 patterns from the 9 observed potential patterns [2]. In BSPM [3,4], the observation range can be extended to the entire chest based on the chest electrodes used in the 12-lead ECG. Therefore, BSPM is more comprehensive and site-specific for analyzing ECG information than the standard 12-lead ECG. Although the electrode arrangements in BSPM provide ample ECG information, they require many electrodes and wiring, burdening the patient and technicians for proper electrode placement. Therefore, BSPM is rarely used in clinical practice [5].

ECG interindividual variability and waveform distortion affect diagnostic accuracy. Waveform distortion, an important issue in ECG diagnostic accuracy, is primarily affected by physiological factors, including differences in conduction velocity, Purkinje fibers, and ionic currents in various parts of the heart [6], as well as geometrical factors, including individual differences in the heart’s size and orientation and physique, age, and sex [7,8]. While the ECG waveforms mainly vary based on individual differences in the heart’s geometry [9], the distance between the electrodes and the heart strongly influences their amplitude [10]. A reliable measurement of the ECG waveform is crucial for diagnosis [5,11,12]. Correct placement of electrodes is challenging, even for well-trained and careful technicians. Positioning an electrode with an error of <1 cm is only 40–50%, whereas misplacement is at 2–3 cm [13]. Several studies have investigated individual differences [7] and electrode misalignment [13] in conventional electrode placements for the 12-lead ECG.

Recently, wearable devices have been used to obtain long-term ECG monitoring for healthcare in our daily lives [14,15]. Wearable devices may provide a promising alternative solution for measuring heart rate variability [14]; however, many issues remain to be resolved regarding the accuracy of ECG measurements required for diagnosis. For these applications, the number of electrodes must be reduced for comfort and portability, such as for bipolar ECG measurements, in which two electrodes are attached to the chest. ECG measurements with fewer electrodes are less robust than those with 12-lead or BSPM because of the reduced number of ECG signals. Furthermore, the electrodes should be closely arranged to reduce the device’s size and weight. However, decreasing the electrode distance lowers the ECG signal amplitude, resulting in a low signal-to-noise ratio. Therefore, electrode positions that maximize the signal amplitude have been investigated [16,17]. From the measurements of three subjects, electrode position in the range of the conventional 12-lead electrodes was suggested. In addition, the amplitude of the ECG signal was maximized when the electrode pair was positioned along the cardiac axis. In experiments, however, it is not straightforward to evaluate individual variability and misalignment separately, in addition to the limitation of the number of electrodes.

A computational approach [18,19,20] may be more promising for the assessment of electrode placement and interindividual variability. However, the interindividual variability of the ECG amplitude and waveform has not been computationally replicated. This could be because most computational approaches are based on finite element methods (FEMs) that use a tetrahedral mesh to model the human body. The implementation of the tetrahedral mesh makes it difficult to fully account for the morphological differences between individuals. We previously developed a computational method to replicate the ECG signal by combining the scalar-potential finite-difference (SPFD) method and time-domain signal processing with validation [21,22]. The proposed method, which can use a voxelized human model, enables the evaluation of ECG signals considering interindividual morphological differences.

In this study, we aimed to clarify the effect of geometrical factors involving the body and heart on the robustness of ECG using different human body models. A morphing technique was used to represent the anatomical variability in the size and orientation of the body and heart. The effect of geometrical factors on the ECG waveform was then examined using the time-series data dissimilarity evaluation method. Furthermore, the placement of ECG electrodes such that the amplitude of the ECG signal is increased and interindividual variability is reduced even with misaligned electrodes was provided.

## 2. Materials and Methods

### 2.1. Anatomical Human Model

We used an anatomical numerical human body model named 4D extended cardiac-torso (XCAT) phantom, which was developed at Johns Hopkins University [23]. XCAT is a voxel model based on computed tomography images, comprising 21 types of biological tissues, including the skin, muscles, fat, bone, and heart. The electrical conductivity of these tissues, excluding the skin, was assigned based on the 4-Cole–Cole dispersion model [24] and that of the skin was set to 0.1 S/m [25].

**Figure 1:**
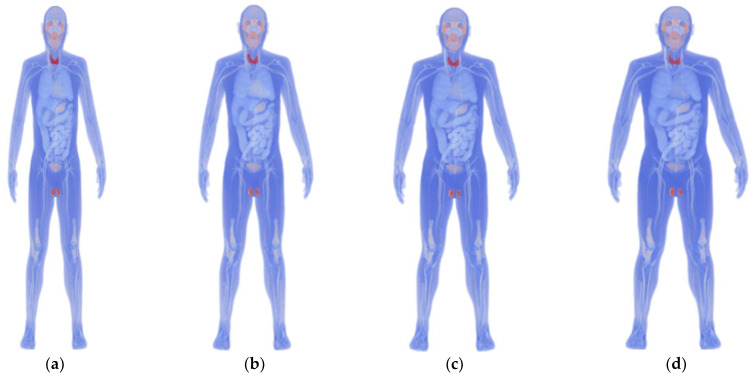
Anatomical numerical human body models developed using the 4D extended cardiac-torso (XCAT) phantom representing (**a**) underweight (BMI: 17.2), (**b**) normal (BMI: 23.2), (**c**) pre-obese (original) (BMI: 28.5), and (**d**) obese (BMI: 34.2).

We used the original XCAT model to develop four models with different body mass indices (BMIs) using a morphing technique. These models represent individual differences in physique and heart that affect geometrical factors (Figure 1) [7,8]. Based on the categories described by the World Health Organization, the BMI values for the models were set to 17.2, 23.2, 28.5, and 34.2 to represent “Under Weight” (BMI < 18.5), “Normal” (BMI: 18.5–25), “Pre-obese (original)” (BMI: 25–30), and “Obese” (BMI > 30). Each model’s height was standardized at 175.2 cm, and variations in heart size between models were maintained within ±10% [26].

**Figure 2:**
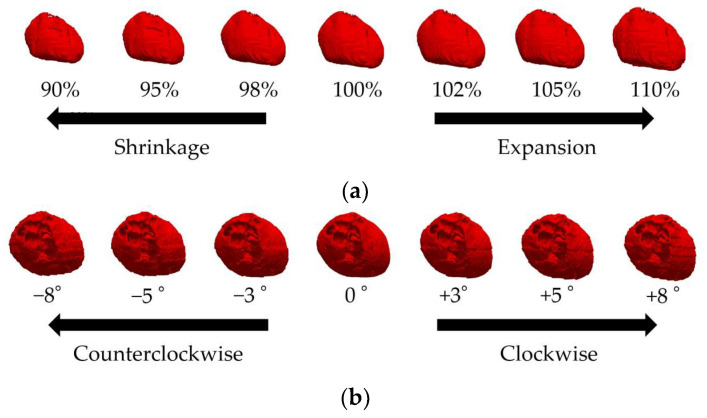
Variations in the XCAT cardiac model showing (**a**) 90%, 95%, 98%, 100%, 102%, 105%, and 110% of the original model volume (front view of the body) and (**b**) −8, −5, −3, 0, +3, +5, and +8 degree rotations (above view of the head) from left to right.

Given that the heart’s size and orientation also contribute to interindividual differences, we scaled the heart by 90–110% using seven scaling models with affine transformation [26] (Figure 2a). Note that the same scaling factor was used for each heart part, and thus chamber size was not tuned, because of the lack of detailed knowledge needed for the model development. Similarly, seven rotation models [27], in which the heart was rotated by −8 to +8 degrees [27], were used to represent the individual differences in the heart’s size and orientation (Figure 2b). The original model for individual differences in the heart’s orientation and size was the “Pre-obese” model described above. The heart’s potential vacant volume, created after affine transformation, was filled with lung tissue.

### 2.2. Bipolar Electrode Pairs

**Figure 3:**
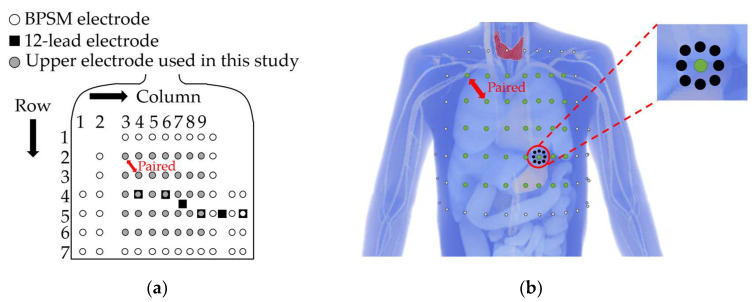
(**a**) Definition of the upper bipolar electrode positions (shown in gray) for evaluating the effect of geometrical factors. (**b**) Electrode misalignment in eight directions with 2 cm: white, black, and green dots indicate original BSPM electrodes, misaligned electrodes displaced by 2 cm, and the upper electrode used as a reference point, respectively.

Bipolar electrode pairs were considered based on the precordium and lateral chest electrodes in BSPM [16] as a fundamental component. Based on the findings of previous studies [16,17], which stated that electrode pairs oriented along the cardiac axis maximize the ECG amplitude, we focused only on the diagonal electrode pairs at an interelectrode distance of approximately 6 cm, the shortest diagonal distance from the BSPM electrodes. The upper electrodes were placed on the left precordial area (rows 2–6 and columns 3–9), and the corresponding lower electrodes were placed diagonally from the upper electrodes meeting the following conditions: when the upper electrode is placed on row 2–column 3, the lower electrode is placed on row 3–column 4 (see red two-headed arrow in Figure 3a,b). The sensitivity of ECG on the bipolar electrode placement lead was examined using 35 (5 × 7) bipolar electrode pairs. The ECG potential in each bipolar lead was calculated as the difference between the two electrodes. In addition to the physical individual differences described in the previous section, we discussed the effects of electrode misalignment on bipolar ECG. Electrode misalignment is expected in ECG measurements [13]. Figure 3b illustrates the positions of the electrode misalignment in eight directions from the reference electrode.

### 2.3. Scalar-Potential Finite-Difference Methods

Because the frequency of the ECG waveform is approximately 1 Hz, the displacement current can be ignored [1,24]. In ECG modeling, different computational methods are used, including electro-quasi-static FEM with tetrahedrons [28]. However, analyzing a whole human body model using FEM is extremely expensive when discretized with tetrahedrons. The SPFD method [29] was used to perform fast computations of the electric field in a voxel body model in the frequency domain [21]. The scalar potential *ϕ*, induced in the volume conductor, is given by the Poisson equation as follows:

(1) $$\{\begin{array}{c} \nabla \cdot \left(\sigma \nabla \phi \right)=-\nabla \cdot J\;\mathrm{in}\;\Omega \;\\ \left(\sigma \nabla \phi \right)\cdot n_{B}=0\;\mathrm{on}\;B_{S} \end{array},$$

where *σ*, ***J***, ***n****_B_*, *Ω*, and *B_S_* are the electrical conductivity, current density, unit vector outwardly normal to the body surface, volume conductor, and body surface, respectively. Equation (1) was discretized on the basis of a quasi-static approximation [25] to obtain the following equation:

(2) $$\left(\sum_{n=1}^{6}S_{n}\right)\phi_{0}-\sum_{n=1}^{6}S_{n}\phi_{n}=-j\omega q,$$

where *n*, *ϕ_n_*, *q*, and *S_n_* denote the node index, electric scalar potential at the *n*-th node, angular frequency, electrical charge at the 0-th node, and edge conductance from the *n*-th node to the 0-th node derived from the tissue conductivity of the surrounding voxels, respectively.

The boundary condition of (2) was satisfied by setting air voxels adjacent to the body surface. Equation matrices were obtained using Kirchhoff’s current law for all nodes. Potentials were solved iteratively using the successive over-relaxation method [27] with a multigrid method as a preconditioner [28]. Six multigrid levels were used to reduce the time required for iterative calculations. The computations were conducted until the relative residual was <10^−6^.

### 2.4. Quasi-Static Finite-Difference Time-Domain Methods

The finite-difference time-domain (FDTD) method is a widely used electromagnetic field analysis method. In FDTD analysis at low frequencies, many analysis steps are required until the body’s magnetic field becomes stable. The Maxwell Equation (3) used in the theoretical equation of the FDTD method can be expressed as Equation (4), considering that the displacement current is negligible in the low-frequency region.

(3) $$\nabla \times H=j\omega \varepsilon \left(1+\frac{\sigma }{j\omega \varepsilon }\right)E,$$

(4) ∇×H=σE,

However, the quasi-static approximation can be used when the wavelength is sufficiently large relative to the size of the human body. The quasi-static FDTD method [30] is then used to reduce the analysis cost through quasi-static approximation. In this study, the quasi-static FDTD method was used to confirm the validity of the SPFD analysis results.

### 2.5. Modeling Cardiac Potentials and Construction of the ECG

**Figure 4:**
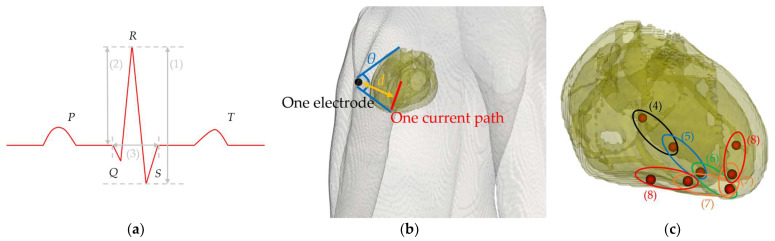
(**a**) ECG outline and the parameter names for waveform characteristics. (1) SA (signal amplitude), (2) R-wave amplitude, and (3) QRS width. (**b**) Visualized image of *d* (heart–electrode distance) and *θ* (solid angle of the heart viewed from the electrode) formed by one electrode and one current path. The two blue lines extend from the electrode’s center and connect to the uppermost and lowermost points of the myocardial tissue in the model. The yellow line represents the distance between the electrode’s center and the current path. (**c**) Positions of the electric dipoles in the heart of the anatomical model that sequentially propagate from (4) to (8). (4) A–V node to bundle of His, (5) bundle of His to bundle branches, (6) bundle branches, and (7) and (8) Purkinje fibers.

Signal propagation in myocardial tissue and the calculation of cardiac potentials using a dipole model are discussed in this section. The method for constructing an ECG using the cardiac potential calculation results is also explained. The cardiac action potentials generated at the sinus node around the superior vena cava in the right atrium [1] sequentially propagate through the myocardial atrium and atrioventricular node (A–V node). This propagation generates cardiac potentials and three typical ECG features, the P, QRS, and T waves [1], corresponding to atrial depolarization, ventricular depolarization, and repolarization, respectively. Figure 4a shows the names of the P to T waves mentioned above and the electrocardiogram parameters used in this study. We computationally replicated only the QRS wave, which has the largest potential and is used to calculate the heart rate and diagnose an arrhythmia. Figure 4b shows the location relationship between the heart, one propagation path, and an electrode. It defines the heart–electrode distance and the heart’s solid angle viewed from the electrode based on the heart’s position and the electrode. In Figure 4b, *d* indicates the electrode–heart distance and *θ* indicates solid angle. The value of *d* at one electrode is calculated from the average of the current paths from (4) to (8). The mechanism of generating the myocardial action potential was simulated by sequentially placing single electric dipoles [22] in the stimulated conduction system of the heart, as validated previously [21,22]. The length of each electric dipole was 2 mm, and the dominant frequency component of the myocardial action potential was 1 Hz.

Figure 4c illustrates the arrangement pattern of the electric dipoles. The dipole moment length in the voxel human model could not be unified because the charges of the input sources are placed only at each vertex. Therefore, the dipole moment was adjusted by introducing a correction factor to the scalar potential obtained from numerical calculations [21], as shown in (5).

(5) $$\phi_{n}^{\prime }\;=\;\phi_{n}\;\times \;\frac{l_{mean}}{l_{dn}},$$

where $\phi_{n}$, $l_{mean}$, and $l_{dn}$ indicate the scalar potential calculated at each dipole, the average dipole length of each part, and the dipole lengths from each analysis, respectively.

ECG was performed using the following procedure based on our previous studies [21,22]: The surface potential of the anatomical human body model was calculated using the SPFD method with a single electric dipole as the input source. Next, the propagation time of the cardiac action potential was calculated using the propagation velocity and the distance between successive charges. The above operations were then repeated for each electric dipole. Finally, the body surface potential distribution at each electric dipole was integrated into the time domain to construct the ECG.

The time series at the *i-*th charge was derived using Equations (6) and (7).

(6) $$t_{i}=\sum_{k=1}^{i}\Delta \;t_{k},$$

(7) $$\Delta \;t_{i}=\left(\frac{\left|r_{i}-r_{i-1}\right|}{v_{part}}\;\;\;\;i=1,2,\ldots ,n\right),$$

where $r_{i}$ and $v_{part}$ indicate the position vector of the *i-*th charge and the propagation velocity in the ventricles, respectively. The propagation velocities in the bundle of His, bundle of branches, and Purkinje fibers, which are the propagation pathways of the myocardial action potentials in the ventricle, were set as 1.25, 1.25, and 3.25 m/s, respectively [1] (see also Figure 4c).

### 2.6. Dynamic Time Warping Methods

For ECG measurements, proper electrode placement enables high amplitude and robustness of the signal [13,16,31], which can be evaluated with individual variations in cardiac potential due to geometrical factors, including individual differences and electrode misalignment. The ECG SA was calculated using Equation (8) as follows:

(8) $$SA=\left|V_{max}-V_{min}\right|,$$

where *V_max_* and *V_min_* represent the maximum and minimum values of the QRS wave, respectively.

Robustness was evaluated using the amplitude and waveform variability of the ECG signals, which are used to automatically diagnose arrhythmia [32,33]. Although variations in ECG signals due to geometrical factors have been previously quantified [8,9,13], no general metrics exist for quantifying their interindividual variability. Only a few methods can be applied to time-series data of different lengths, depending on the individual and measurement conditions. Therefore, we used an index of dynamic time warping (DTW) [34,35] to evaluate the dissimilarity between the two time-series data of different lengths, such as ECG, by stretching or shrinking the time axis [35]. Given the two different lengths of the time-series data *S* and *T* in (9), the dissimilarity DTW was calculated using Equation (10).

(9) $$\begin{array}{l} S=\left\{s_{1},s_{2},\cdots ,s_{i},\cdots ,s_{n}\right\}\\ T=\left\{t_{1},t_{2},\cdots ,t_{j},\cdots ,t_{m}\right\} \end{array},$$

(10) $$DTW(S,T)=\sum_{k=1}^{n}\delta \left(\omega_{k}\right)=\sum_{k=1}^{n}\left|s_{i_{k}}-t_{j_{k}}\right|,$$

where *s_i_* and *t_j_* represent the coordinate data and each subscript represents the order of the observed time. *δ*(*ω_k_*) represent the distance between the points associated with the stretching transformation, and *i_k_* and *j_k_* represent the indices of *S* and *T* associated with the stretching transformation, respectively.

The variation in the waveform was evaluated by normalizing the ECG signal on the basis of the following equation and calculating the dissimilarity DTW on the basis of Equation (10):

(11) $$V^{\prime }(t)=\left(V(t)-V_{\min}\right)/\left|V_{\max}-V_{\min}\right|,$$

where *t* represents the time of the ECG signals.

DTW and its normalized value might be considered metrics of variation in ECG amplitude and waveform (shape), respectively.

### 2.7. Multivariate Analysis

Multivariate analysis [36] is used to statistically analyze the relationship between multiple independent variables and a single dependent variable. Unlike single regression analysis, which examines one dependent variable for one independent variable, multivariate analysis evaluates how multiple independent variables affect the dependent variable. We investigated the correlation of DTW and normalized DTW with the superposition of the electrode–heart distance and solid angle using JMP 16 software (SAS Institute, Cary, NC, USA). Statistical significance was set at *p* < 0.05, and the variance inflation factor (VIF) was set at VIF < 10 for no multicollinearity. The first factor, discussed in a previous study [10], may be related to ECG amplitude. The latter factor was introduced in this study as a geometrical factor that approximately provides the whole picture of the heart in combination with the first factor.

### 2.8. Evaluation Procedure

The amplitude and waveform (shape) variations in the ECG signal due to individual differences were evaluated using the following procedure: First, we reconstructed the ECG signal by placing the bipolar electrodes at positions V_1_ to V_6_ of a 12-lead ECG and compared it with the measured data [37]. Then, we computationally constructed an ECG waveform for 35 different bipolar lead combinations to evaluate individual differences in body size (*N* = 4, Figure 1), heart size (*N* = 7, Figure 2a), and orientation (*N* = 7, Figure 2b).

DTW was calculated for the *_N_*C_2_ pattern, which is a combination of *N* different human body models. The mean DTW for all combinations was calculated to represent the individual differences in the ECG amplitude for the corresponding bipolar lead combination. This procedure was repeated for different body and heart sizes and heart orientations. The same method was used to calculate the normalized DTW, an index of ECG waveform variability.

The ECG waveform was computed for the bipolar leads with electrodes shifted by 1 cm and 2 cm [13] in eight directions from the reference position (Figure 3b). Furthermore, electrode misalignment in the bipolar leads was considered using two patterns, representing misalignments in either the upper or lower electrode. The DTW method was applied to the ECG waveform for one bipolar lead with a correctly positioned electrode, and the misalignment of that electrode in a specific direction was evaluated. Eight DTW combinations were obtained (Figure 3b). The mean of these DTW values (16 patterns) was calculated as the variability of the ECG amplitude due to electrode misalignment. The normalized DTW was also calculated.

## 3. Results

### 3.1. Verification in the Construction of the ECG Waveform Using the 12-Lead ECG

**Figure 5:**
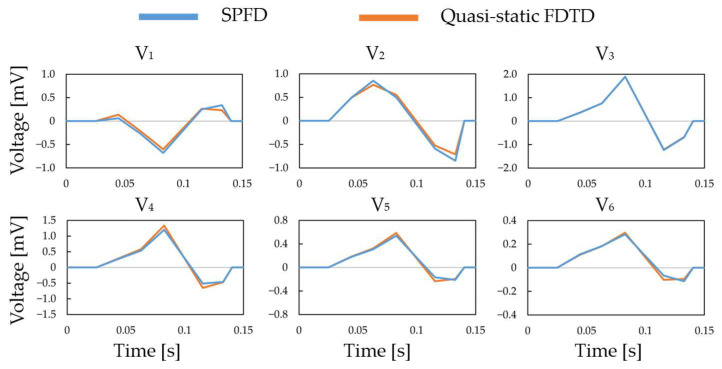
QRS waves in the chest leads calculated using the SPFD and quasi-static FDTD methods.

**Figure 6:**
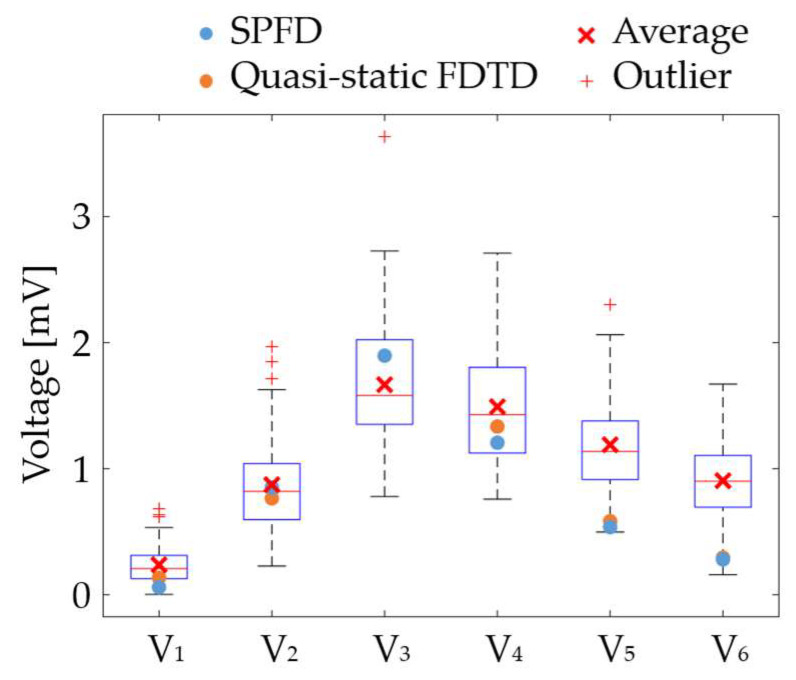
Comparison of the computed and measured R-wave amplitudes.

The effectiveness of the proposed ECG generation method with SPFD was verified by comparison with the computation with quasi-static FDTD. For the original XCAT model, the ECG waveforms obtained at different chest leads (electrode positions) are shown in Figure 5. The PRD (percent root mean square difference) of the ECG calculated by the SPFD method relative to that with the quasi-static FDTD method are 22.0, 13.2, 0.99, 11.3, 11.2, and 11.2% for V_1_ to V_6_, respectively. For further verification, our computational results were compared with measured ECG data [37] in terms of the R-wave amplitude (refer to Figure 5a) at each chest lead (V_1_ to V_6_). Figure 6 shows the box plot diagram comparing the R-wave amplitude for the measured values. Furthermore, the computed value for the original XCAT model is also shown for comparison. Because the R-wave amplitude of each chest lead differs greatly depending on the transition zone, the measured data for 74 subjects were used with a transition zone at V_2_ and the maximum R-wave amplitude at V_3_. Figure 6 shows the computed amplitude of the R wave at different electrode positions, together with the measured results for the 74 subjects. Computational values were generally within the quartile range. QRS width (Figure 4a) was 0.115 [s] in the computation, whereas it ranged from 0.058 to 0.126 [s] in the measured waveforms.

### 3.2. Validation in the Signal Amplitude of the QRS Wave

**Figure 7:**
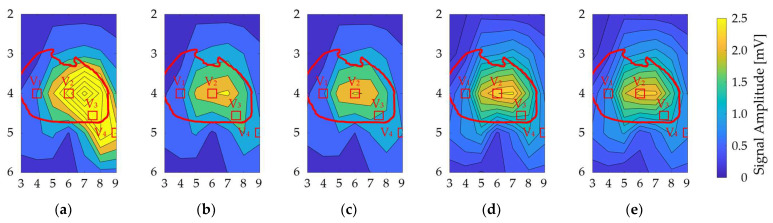
Contour plot of the ECG signal amplitude variability for different bipolar electrodes. The vertical and horizontal axes correspond to the rows and columns in Figure 3a. There are variations due to (**a**) physique, (**b**) heart size, (**c**) heart orientation, and (**d**,**e**) misalignment by 1 cm and 2 cm, respectively. The red oval corresponds to the heart’s position when the human body is viewed from the front. The red square indicates the position of the chest electrode in the standard 12-lead ECG as a reference.

The ECG signal amplitude in the bipolar ECG (35 pairs) averaged over all models: *N* = 4 for the physique (6 simulations), *N* = 7 for the heart’s size and orientation (21 simulations each), and *N* = 8 for each upper and lower electrodes (16 simulations) for separations of 1 cm and 2 cm (Figure 7). The oval-shaped red line corresponds to the heart’s position when the human body is viewed from the front. The signal amplitude reached a maximum around V_2_ and V_3_ for different geometrical factors, as observed for columns 6, 7, and 8 in row 4 of the BSPM electrode positions (Figure 7).

### 3.3. Variation in DTW and Normalized DTW

**Figure 8:**
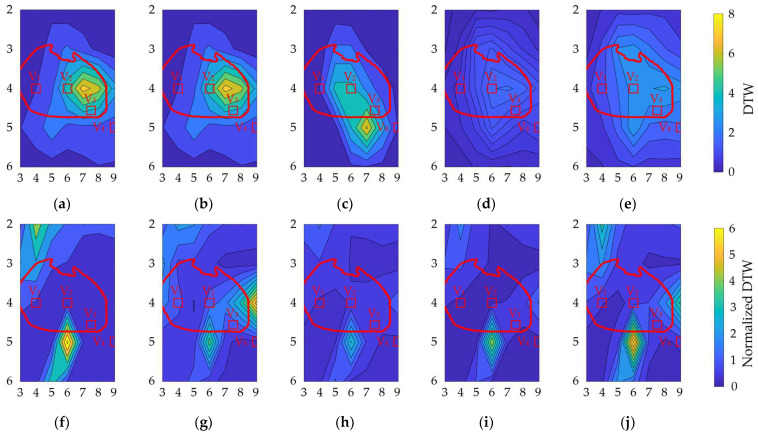
Contour plot of (**a**–**e**) DTW and (**f**–**j**) normalized DTW for different bipolar electrodes. Variations due to (**a**,**f**) physique, (**b**,**g**) heart size, (**c**,**h**) heart orientation, and (**d**,**i**) and (**e**,**j**) misalignment by 1 cm and 2 cm, respectively. The vertical and horizontal axes correspond to the rows and columns in Figure 3a. The red oval corresponds to the heart’s position when the human body is viewed from the front. The red square indicates the position of the chest electrode in the standard 12-lead ECG as a reference.

Figure 8 shows the contour plots of the variability in DTW and normalized DTW for each geometrical factor, showing higher DTW on the left chest of V_2_. These electrode positions have higher amplitude values, indicating a trade-off between amplitude and waveform variations in bipolar ECG measurements. However, within row 4, the signal amplitude variation in column 6 was lower than that in columns 7 and 8. The distributions of misalignment were more spread out. For normalized DTW, high and low values were observed at high SA in the lower part of V_2_ and row 4, respectively.

### 3.4. Statistical Analysis of the Geometrical Factors

The influence of two geometric factors, i.e., heart–electrode distance and solid angle between cardiac electrodes, on the ECG signal was evaluated. The difference between the heart–electrode distance and the solid angle was calculated for all 35 electrode pairs in each model. The average of these metrics for the 35 electrode pairs was defined as the difference in heart–electrode distance and solid angle between the two models. As explained in Section 2.8, for the physique, scaling, and rotation models, there were 48 patterns of DTW and normalized DTW (6, 21, and 21 patterns, respectively). Given that DTW and normalized DTW evaluate the difference between the two indices, 48 patterns of heart–electrode distance and solid angle are obtained for each index.

First, a linear regression analysis was conducted for the heart–electrode distance and solid angle differences as independent variables and DTW and normalized DTW as objective variables. The results show statistically significant *p*-values: *p* < 0.0001, *p* = 0.031, *p* < 0.0001, and *p* < 0.0005 for the correlations between DTW and distance difference, DTW and angle difference, normalized DTW and distance difference, and normalized DTW and angle difference, respectively.

We then performed a multivariate linear regression analysis based on these results. The VIF for the heart–electrode distance and solid angle differences was 1.09 (<10), suggesting no multicollinearity between the heart–electrode distance and solid angle differences. The contribution rates of the heart–electrode distance and solid angle differences in DTW were 92.6% and 7.4%, respectively. The contributions of the heart–electrode distance and solid angle differences in normalized DTW were 33.7% and 66.3%, respectively.

### 3.5. Optimal Position of the Electrodes

**Figure 9:**
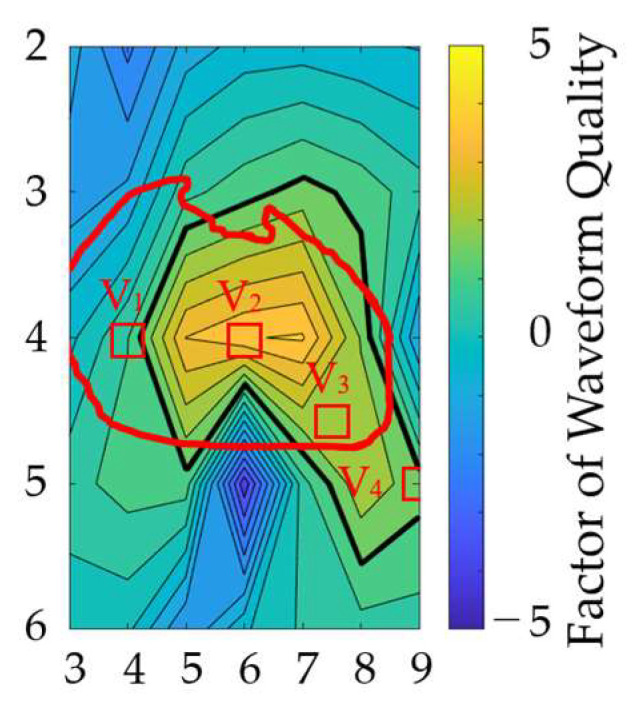
Electrode attachment area, which is insensitive to geometric and misalignment factors. The vertical and horizontal axes correspond to the rows and columns in Figure 3a. The thick black line indicates the region of electrode positions for the top 20% of the waveform quality factors. The red oval corresponds to the heart’s position when the human body is viewed from the front. The red square indicates the position of the chest electrode in the standard 12-lead ECG as a reference.

The locations of the bipolar electrodes that provide large-amplitude and less waveform distortion were evaluated in terms of the SA and normalized DTW. The SA and normalized DTW were normalized using the maximum value for each individual difference and electrode misalignment, and then their differences were defined as a factor of waveform quality. Figure 9 shows the factor of waveform quality computed from the results in Figure 7 and Figure 8b. The electrode positions of V_1_ to V_4_ in the standard 12-lead ECG are included in the region of electrode positions for the top 20% of the waveform quality.

## 4. Discussion

This study computationally evaluated the individual variability in the amplitude and shape of the QRS wave for bipolar lead pairs. Electrode misalignment by a typical technician was demonstrated that an error of <1 cm was only 40–50% [13]. The uncertainty of the measurement and electrode misalignment necessitated computational evaluation. To investigate this variability, we considered 48 scenarios using 35 bipolar electrode pairs (1680 waveforms) in anatomical human body models.

As shown Figure 7, the signal amplitude was high for the V_2_ and V_3_ electrode positions for different geometrical factors, corresponding to the heart. As in a previous study [16], the signal amplitude was larger in row 4. The most influential geometrical factor was the model’s physique. Because of the electrode–heart distance, the amplitude was high for the underweight and normal models, with average values of 2.05 mV and 1.2 mV, respectively. The highest amplitudes were observed for columns 6, 7, and 8 in row 4 of the BSPM electrode positions.

DTW was higher on the left chest of V_2_ for the considered geometrical factors (Figure 8). In contrast, a higher normalized DTW appeared approximately 6 cm below V_2_ for all geometrical factors, except heart size. These electrode positions exhibited higher amplitude values, indicating a trade-off between amplitude and waveform variations in the bipolar ECG. However, within row 4, the signal amplitude variation in column 6 was lower than that in columns 7 and 8 of the BSPM electrode positions. The distributions of misalignment were more spread out. For normalized DTW, high and low values were observed in the lower part of V_2_ and row 4, respectively, for high signal amplitude.

A multivariate analysis confirmed the high contribution of the heart–electrode distance difference to DTW, suggesting that the QRS-wave amplitude and heart–electrode distance are closely related. The high dependence on a solid angle difference for normalized DTW was consistent with a previous study [7], which showed that the solid angle of the cardiac outline viewed from a point on the chest dominates the shape of the ECG.

Furthermore, in terms of the observation of higher SA and lower normalized DTW (the factor of waveform quality), the electrode positions of the bipolar leads which are insensitive to different uncertainty factors were examined. Figure 9 shows that appropriate electrode positions were identified, including four electrode positions in the standard 12-lead ECG. This finding suggests the appropriateness of conventional and potential electrode positions for bipolar ECG. In addition, positions with relatively higher factors would be useful for potential wearable sensing applications.

One limitation of this study is the modeling of the heart, which may influence the results in Figure 8b,f. Different head and heart models were generated using scaling factors reported in the literature. However, the size of each chamber may depend on the age, gender, and race/ethnicity [38]. Even for this limitation, qualitive finding, i.e., the area where the ECG is less sensitive, may not be influenced because our discussion was generalized as much as possible in terms of electrode–heart distance and the solid angle of the heart from the electrode.

## 5. Conclusions

In this study, we evaluated the individual variability in the amplitude and shape of the QRS wave. With large-scale computations using anatomical human body models, the dominant factors that affect them for bipolar lead pairs were assessed. From a statistical analysis, the heart–electrode distance and the solid angle of the heart from the electrode were found to characterize the amplitude and waveform of the ECG signals, respectively. The electrode locations close to the location of leads II and III in the standard 12-lead were the most suitable places for less sensibility. Our findings will facilitate the placement of ECG electrodes for wearable devices and the interpretation of the measured ECG signal. Future studies will discuss its application in diagnosis and wearable monitoring considering intersubject variability.

## Data Availability

Raw data in this study is available based on reasonable request.
